# “I want to get myself as fit as I can and not die just yet” – Perceptions of exercise in people with advanced cancer and cachexia: a qualitative study

**DOI:** 10.1186/s12904-022-00948-x

**Published:** 2022-05-17

**Authors:** Kelcey A. Bland, Meinir Krishnasamy, Evelyn B. Parr, Stella Mulder, Peter Martin, Luc J. C. van Loon, Prue Cormie, Natasha Michael, Eva M. Zopf

**Affiliations:** 1 Mary MacKillop Institute for Health Research, Australian Catholic University, Level 5, 215 Spring St., Melbourne, VIC 3000 Australia; 2 Peter MacCallum Cancer Centre, Melbourne, VIC Australia; 3 Department of Oncology, Sir Peter MacCallum, University of Melbourne, VIC, Australia; 4 Department of Nursing, Faculty of Medicine, Dentistry and Health Sciences, University of Melbourne, Melbourne, VIC Australia; 5 University of New South Wales, Sydney, NSW Australia; 6 School of Medicine, Deakin University, Geelong, VIC Australia; 7 Palliative Care, Barwon Health, Geelong, VIC Australia; 8 Department of Human Biology, NUTRIM School for Nutrition and Translational Research in Metabolism, Maastricht University, Maastricht, Netherlands; 9 School of Medicine, University of Notre Dame, VIC, Australia; 10 The Szalmuk Family Department of Medical Oncology, Cabrini Health, Melbourne, VIC Australia

**Keywords:** Advanced cancer, Palliative care, Cancer cachexia, Exercise, Physical activity, Barriers, Motivators, Preferences, Qualitative interviews, COVID-19 pandemic, Thematic analysis

## Abstract

Cachexia is a prevalent muscle wasting syndrome among people with advanced cancer that profoundly impacts patient quality of life (QoL) and physical function. Exercise can improve QoL, physical function, and overall health in people with cancer and may be an important addition to treatment approaches for cancer cachexia. Greater understanding of patients’ perception of exercise can help elucidate the feasibility of implementing exercise interventions for cancer cachexia and facilitate the design of patient-centered interventions. We aimed to describe the perception of exercise in patients with advanced cancer and cachexia, and capture exercise motivators, barriers, and preferences, to inform the feasibility of exercise interventions. Individual interviews (*n* = 20) with patients with locally advanced or metastatic cancer with cachexia were conducted and analyzed using reflexive thematic analysis. Main themes from interviews were: 1) Life is disrupted by cancer and cachexia; 2) Exercise offers hope; 3) Exercise barriers are multifaceted; and 4) Exercise access and support are important. Participants reported that their cancer and cachexia had intensely altered their lives, including ability to exercise. Exercise was perceived as important and participants described a hope for exercise to improve their health and wellbeing. Yet, several complex exercise barriers, such as burdensome cancer symptoms and the overwhelming impact of the COVID-19 pandemic, hindered exercise participation and prevented participants from fully realizing the perceived benefits of exercise. Factors believed to improve exercise engagement and overcome exercise barriers included increased exercise support (e.g., professional supervision) and accessibility (e.g., convenient locations). Patient-reported exercise barriers and preferences can inform the design of exercise interventions, particularly within future research studies aiming to establish exercise feasibility and efficacy in people with advanced cancer and cachexia.

**Supplementary Information:**

The online version contains supplementary material available at 10.1186/s12904-022-00948-x.

## Introduction

Cancer cachexia is a multifactorial syndrome characterized by the ongoing loss of muscle mass, with or without the loss of fat mass, that cannot be reversed with conventional nutritional support alone and leads to progressive functional impairment [1]. Cachexia is prevalent in all cancer types, but tends to disproportionately affect people with more advanced or incurable disease [2, 3]. Prominent adverse effects of cancer cachexia include increased mortality [4–6], greater treatment toxicity [7, 8], declines in physical function [9, 10], and reduced overall quality of life (QoL) [11–13]. Despite the significant burden cancer cachexia places on both patients and their carers [14, 15], cachexia remains both challenging to clinically identify and treat.

A multimodal intervention strategy that includes pharmaceuticals, dietary support, and structured exercise training may hold the greatest potential to support patients with cancer cachexia, although evidence remains limited [16, 17]. Specific to both aerobic and resistance exercise training, benefits include the possibility for exercise to address the underlying causes of cancer cachexia and the associated adverse effects. On one hand, exercise may help counteract disrupted metabolism, for example, due to chronic inflammation, causing cancer-related weight loss or muscle wasting [18, 19]. Equally, exercise might prevent declines in skeletal muscle mass [20] and manage cancer symptoms, increase physical function, and improve overall QoL [21, 22]. There is evidence that aerobic and resistance exercise training (typically delivered in a structured, supervised setting) is beneficial among people with advanced cancer [23], including older adults with advanced cancer undergoing treatment [24]. Preliminary evidence from a recent randomized controlled trial in 40 patients with pancreatic cancer and cachexia also found a three month, supervised resistance training intervention may improve muscle strength and lean body mass [25]. However, more information on the feasibility and efficacy of exercise as either a stand-alone or as part of a multimodal strategy for the management of cancer cachexia, including among patients with advanced or incurable disease, is needed.

A critical first step in establishing the feasibility of exercise for patients with advanced cancer and cachexia includes exploring patients’ openness and willingness to exercise. Adverse effects of cancer cachexia, including a loss of appetite, reduced food intake, and involuntary body weight loss [26, 27] may exacerbate cancer symptoms and reduce physical function and strength [28–30]. Thus, the burden of both an advanced or incurable cancer diagnosis coupled with the experience of living with cachexia may reduce exercise motivation and tolerability relative to other people with cancer. Several qualitative studies report that people with advanced cancer experience complex barriers to exercise, including cancer-specific (e.g., cancer-related fatigue), psychosocial (e.g., low confidence or mood), and environmental factors (e.g., weather) [31–35]. Despite these reported challenges, patients with advanced cancer still express interest in exercise and view exercise positively [31–34, 36]. To our knowledge, perceptions of exercise in those with advanced disease who have cancer cachexia have not been specifically investigated. Understanding patients’ experiences with and interest in exercise is key to establishing the feasibility of implementing exercise as a meaningful intervention for cancer cachexia. The current descriptive qualitative study aimed to explore the perceptions of exercise among patients living with advanced cancer and cachexia and capture perceived exercise motivators, barriers, and preferences.

## Materials and methods

### Study design

We conducted a descriptive qualitative study using individual, semi-structured interviews. Reflexive thematic analysis was applied to the data to understand perceptions of exercise among people with advanced cancer and cachexia. Quantitative data on participant demographics and medical characteristics, cachexia and nutritional status, and current physical activity levels were collected to provide context and to aid the transferability of the study’s qualitative findings. The reporting of this study is in accordance with the Standards for Reporting Qualitative Research (SRQR) checklist [37].

### Participants

Eligible participants were adults (≥ 18 years) with metastatic or locally advanced cancer (e.g., unresectable cancer or a larger tumor that has spread to nearby lymph nodes or tissues) with cachexia (i.e., involuntary body weight loss > 5% over the previous six months; or weight loss > 2% and body mass index (BMI) < 20 kg/m^2^) [1]. Participants were excluded if they had an expected survival of < 3 months, were receiving parenteral nutrition or enteral nutrition via a feeding tube, were less than four weeks post-surgery, had full-time reliance on a mobility aid (e.g., wheelchair) for all day-to-day activities, or were unable to communicate in English. Patients were recruited via convenience sampling. Patients were referred to the study by a member of their cancer care team, including palliative care physician or medical oncologist, from St. Vincent’s Hospital Melbourne, Cabrini Health, and Barwon Health in Victoria, Australia or learned about the study via word-of-mouth from community-based clinicians. A target sample size of 20 participants was deemed sufficient to support data adequacy in terms of the number and variety of participant experiences that would be collected. Informed consent was obtained from all participants. Participants were interviewed between July 2020 and April 2021 and thus, following the commencement of the coronavirus (COVID-19) pandemic and often during periods of public health restrictions in Victoria, Australia.

### Data collection

A single 30-min one-on-one semi-structured interview was conducted over the phone or via a videoconference call. All participants completed the interviews from home in a private space. Interviews were conducted by a cisgender woman, doctoral research student, and exercise physiologist (KB), with prior graduate-level training in qualitative research methods. No interviewees had previously worked with the interviewer in an exercise setting. Participants did, however, have an awareness of the interviewer’s professional and research experience in exercise oncology. The interview guide was informed by prior research and the clinical expertise of study team members (KB, MK, and EZ). Interview follow-up questions and prompts were employed to facilitate discussion and elicit more detailed responses. The interview guide was pilot tested with a non-study team member with relevant clinical and research experience and revised, accordingly. During the interview, participants were asked to discuss their experiences and perceptions of physical activity, including both incidental physical activity (e.g., occupational, household) and planned, structured and repetitive physical activity for the purpose of maintaining physical fitness, i.e. exercise [38]. Discussions aimed to capture participants’ overall current physical capabilities, including any changes in physical function since being diagnosed with cancer, as well as their tolerance for and interest in exercise, specifically. The final interview guide is provided is Table 1.

**Table 1:** Interview Guide

Main Questions and Prompts
1. Do you currently do any type of exercise or physical activity? • If yes, please describe the type of activity. a) structured exercise (e.g., gym), b) other leisure-based (e.g., sports), c) household-based (e.g., gardening), d) transportation (e.g., walking to train) and e) occupational • If no, please describe why. a) no previous exercise or activity, b) lack of interest in exercise, c) exercise not enjoyable, d) don’t see point of exercise, and/or d) injury or illness related
2. Has your cancer diagnosis or cancer treatments affected your daily level of activity or ability to move? • Please describe any changes. a) type of change and b) when change occurred
3. Is it important for you to be able to be physically active? • a) perceived benefits, b) perceived harms and c) what does being physically active mean for you (physically, mentally, emotionally, socially)? • If unable to be physically active, what does this mean for you (physically, mentally, emotionally, socially)?
4. What (if anything) makes it difficult for you to stay physically active or to exercise in your current situation? • a) cancer symptoms, b) motivation, c) safety, d) other injuries or illnesses, d) logistics (e.g., travel) and e) COVID
5. Is there anything that does- or might motivate you to be physically active or exercise? • a) advice or information about exercise, b) perceived benefits of exercise, b) certain types of exercise, c) people to exercise with, d) structured program or professional supervision, and e) access
6. Has anyone spoken with you about being active or engaging in exercise following your cancer diagnosis? • If yes, who and what did you speak about? a) reasons provided to exercise or b) advice not to exercise or concerns • If no, would you have wanted to speak with somebody about exercise? Please describe.
7. I am interested in hearing your thoughts about what exercise you might prefer. • a) setting (e.g., outdoors, fitness centers, home), b) type (e.g., walking, strength training, yoga) and c) time (e.g., duration, time of day) • Do you prefer exercising on your own or with others? If with others, who and why?
8. Any other areas/things you’d like to mention or discuss?

Participant demographics were collected using a researcher-generated questionnaire, along with relevant medical information, including current cancer diagnosis, stage and treatment. The Patient-Generated Subjective Global Assessment Short Form (PG-SGA SF) was administered to characterize nutritional status. The PG-SGA collects patient body weight, food intake, nutritional impact symptoms (e.g., nausea, vomiting, lack of appetite), functional capacity, metabolic demands, and includes a physical assessment [39]. The short form version of the PG-SGA has been validated in oncology outpatients [40, 41] and forgoes the disease/condition, metabolic demands, and physical assessment components, so that it may be completed entirely by the patient. Total point scores for the PG-SGA SF were calculated to determine malnutrition severity [40]. A modified-version of the Godin Shepard Leisure Time Questionnaire was used to collect patient-reported physical activity levels, including the number of times they completed mild, moderate, and strenuous aerobic exercise and any resistance exercise training within a typical 7-day period [42]. A Leisure Score Index was calculated for aerobic exercise (frequency of mild × 3, frequency of moderate × 5, and frequency of strenuous × 9); where an index ≥ 24 was classified as being “sufficiently active,” an index of 14–23 as “moderately active,” and an index < 14 as “insufficiently active” [42].

### Data analysis

Descriptive statistics were used to summarize questionnaire data. Quantitative data are presented as totals and percentages or mean ± standard deviation (SD). Given the current study aimed to investigate an under researched construct, qualitative data analysis and interpretation of the interviews was performed using inductive, reflexive thematic analysis by the first author (KB), as described by Braun and Clarke [43, 44]. Reflexive thematic analysis was chosen because of its flexibility and potential to offer rich and complex understandings. A social constructionist approach underpinned the analysis, where meaning and experience are understood to be socially produced [43]. Thus, the thematic analysis took place at the latent level and involved interpretative work. We sought to acknowledge and consider underlying ideas, assumptions, and meanings that shaped what was articulated in the data. All interviews were audiotaped, de-identified and transcribed verbatim. Interview transcripts were cross-checked with audio files and verified for accuracy. Initially, interviews were read and re-read for data familiarization. Two transcripts were also read and reviewed by a blinded study team member (SM). Prior to coding, overarching concepts were mapped out and relevant notes were written down and discussed (KB and SM). An initial coding framework was developed (KB and SM) to allow for systematic coding of data, but not with the intention of pre-defining themes (Supplementary File). Initial coding of the entire data set took place using NVIVO software (version 12, QSR International Pty Ltd) [45] and initial codes were then sorted into meaningful groups for interpretive analysis. Candidate themes were developed and an initial thematic map was created. Any experiences that stood apart from the developing themes were explored further to ensure an accurate representation was provided and themes illustrated data complexity, including any contradictions within the data. Themes were then reviewed and, as appropriate, further refined to ensure data within each theme meaningfully aligned. Subthemes (i.e., themes within themes) were identified to provide further structure and to illustrate a hierarchy of meaning within the data. Theme names, final descriptions, and the allocation of quotes were questioned and critiqued for credibility and trustworthiness by the entire study team over multiple rounds until themes were deemed to provide an accurate representation of the data.

### Researcher reflexivity and rigour

The subjectivity of the first author researcher (KB) was considered a resource during both data collection and analysis [46]. Subjectivity guided decision-making during interviews and informed the flow of questioning and prompts, and the nature and type of verbal response elicited to the often sensitive topics discussed. Participants were listened to with empathy, compassion, and curiosity. Throughout the thematic analysis, interview data were actively interpreted and not merely described, with both researcher and participant subjectivity recognized throughout. The researcher acknowledged, for example, that interviews were conducted in a familiar and comfortable setting to encourage participants to speak more freely about their experiences. Throughout data analysis, the researcher actively reflected on how her experience and knowledge may influence both participant responses and data interpretation, allowing her to identify contradictions within individual participant interviews. Both familiar and new concepts and ideas were identified, noted, and reflected upon during the analysis to allow for a full and complete interpretation of the data.

## Results

### Participant characteristics

Twenty-two patients were invited to be interviewed. One patient declined and another did not take part due to cancer symptoms. Thus, 20 patients were interviewed. Participant characteristics are presented in Table 2. Participants (mean age: 61 ± 13 years) had diverse cancer types. Most patients had metastatic disease and 90% of patients were diagnosed with incurable cancer. Two-thirds of participants were considered critically malnourished (PG-SGA SF scores ≥ 9). Patient-reported physical activity levels indicated that four participants (20%) were sufficiently active (score index: ≥ 24), two participants (10%) were moderately active (score index: 14–23) and 14 participants (70%) were insufficiently active (score index < 14).

**Table 2:** Participant Characteristics

**Demographics**	
Age (years) (mean ± SD)	61 ± 13
Sex (n (%))	
Female	14 (70%)
Male	6 (30%)
** Marital Status (n (%))**	
Single	3 (15%)
Married/Committed Relationship	14 (70%)
Divorced/Separated/Widowed	3 (15%)
** Employment Status (n (%))**	
Retired	11 (55%)
Employed	5 (25%)
Unemployed/Other	4 (20%)
** Education (n (%))**	
Highschool Diploma or Less	4 (20%)
Bachelor’s Degree/Diploma	12 (60%)
Higher than a Bachelor’s Degree	3 (15%)
Prefer not to Answer	1 (5%)
**Medical Characteristics**	
** Disease Stage (n (%))**	
Metastatic	16 (80%)
Locally Advanced	4 (20%)
** Tumor Type (n (%))**	
Gastrointestinal	4 (20%)
Breast	3 (15%)
Lung	2 (10%)
Gynecological	2 (10%)
Sarcoma	2 (10%)
Hematological	2 (10%)
Other	5 (25%)
** Current Treatment (n (%))**	
Chemotherapy	8 (40%)
Immunotherapy	5 (25%)
Hormonal/Targeted Therapy	3 (15%)
Not Undergoing Treatment	4 (20%)
**Cachexia and Nutritional Status**	
** Anthropometrics (mean ± SD)**	
Body Weight (kg)	60.0 ± 8.3
Body Mass Index (kg/m^2^)	21.5 ± 2.4
Six-month Weight Loss (%)	6.0 ± 2.5
** Patient-Generated Subjective Global Assessment Short Form**^a^	
Score 2–8 (n (%))	7 (35%)
Score ≥ 9 (n (%))	13 (65%)
Total Score (mean ± SD)	10 ± 5
**Physical Activity Levels**	
** Godin-Shephard Leisure-Time Physical Activity Questionnaire Leisure Score Index (mean ± SD)**	
Mild	20.3 ± 17.1
Moderate	8.8 ± 13.1
Strenuous	3.1 ± 10.4
Overall_moderate+strenuous_	11.9 ± 18.6
** Resistance Exercise (mean ± SD)**	
Minutes per week	16.3 ± 27.4

^a^Patient-Generated Subjective Global Assessment Short Form categories: 0 to 1: no nutritional problems or need of intervention; 2 to 8: patients with increasing nutritional problems who might benefit from but are not in critical need of clinical interventions; and ≥ 9: critical need for improved symptom management and/or nutrition-intervention options

### Interview themes

**Fig. 1:**
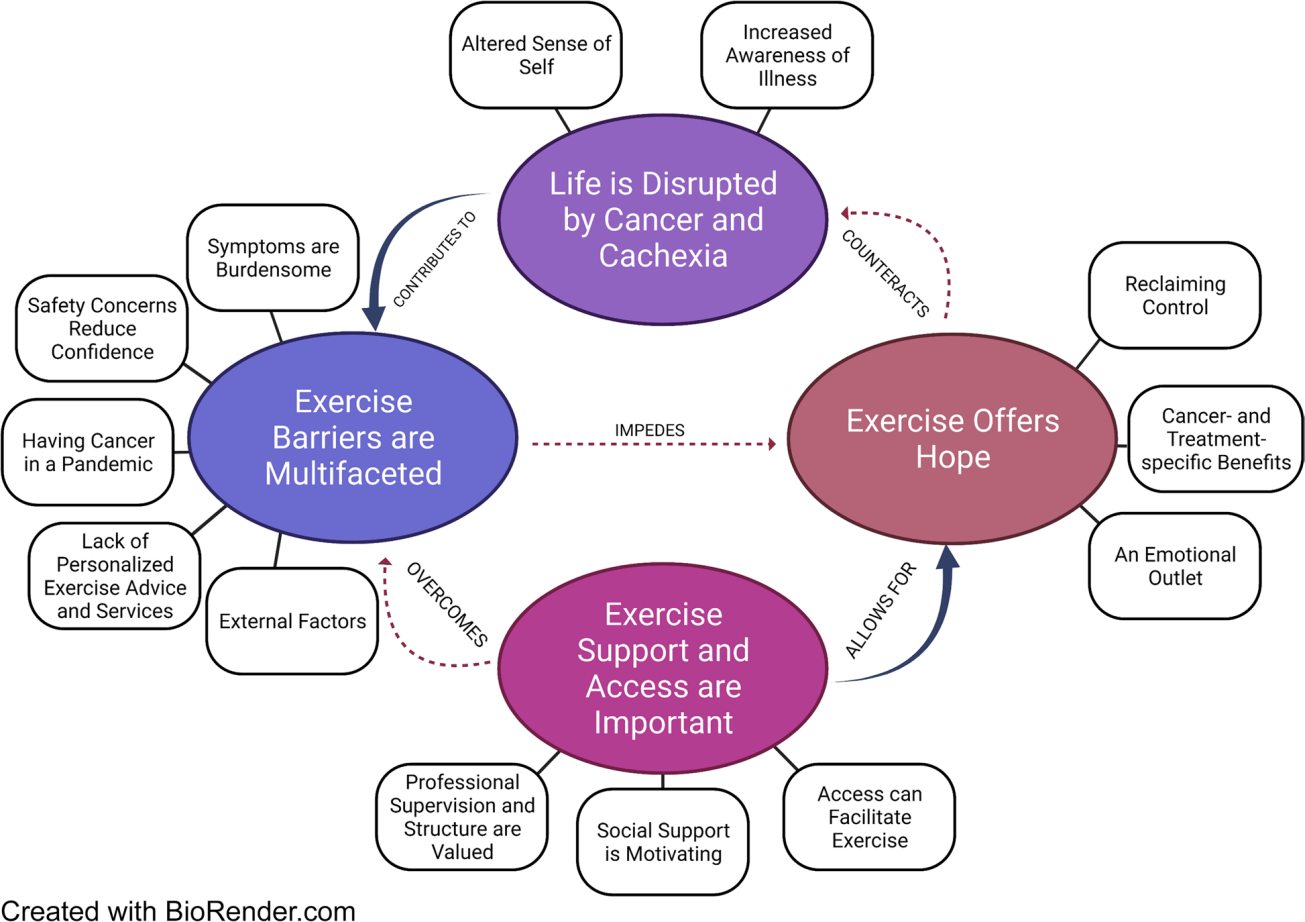
Thematic Map

Reflexive thematic analysis of interview data resulted in the generation of four main themes, each with a series of subthemes (Fig. 1). The first theme, “Life is disrupted by cancer and cachexia,” relates to the extent to which participants felt their cancer diagnosis and cachexia influenced several facets of life, reflecting on changes in physical wellbeing, independence, and activities of daily living, decreased social participation, and reduced mental and emotional wellbeing. The second theme, “Exercise offers hope,” encapsulates how exercise was perceived as a potential tool to overcome the adversity participants were experiencing because of their cancer and cachexia. The third theme, “Exercise barriers are multifaceted,” captures the myriad of exercise barriers preventing participants from fully realizing their believed benefits that exercise held, including barriers directly relating to or exacerbated by their cancer. “Exercise access and support are important” was the fourth and final theme that relates to key factors that participants felt may help to overcome perceived exercise barriers and foster exercise participation in a safe, enjoyable, and more effective way. Themes, subthemes, and representative participant quotes are presented in Table 3 and are described in more detail hereafter.

**Table 3:** Main Themes and Subthemes

Themes and Subthemes	Representative Quotes
**1. Life is Disrupted by Cancer and Cachexia**	
1.1 Altered Sense of Self	I used to do everything. It's affected my thinking, my memory. I'm always tired. I was, I was always busy, busy, busy and now, I don't know how I can feel as if the day's gone and I've done nothing. It's slowed me down. I can't go, I don't go to the shops, not just because of the COVID. But before COVID, I couldn't go to the shops on my own. My husband drives me everywhere. So, I've stopped driving. Yes. So it's just changed from being a very independent person to someone that relies on others. – ID #9
	I don't feel as healthy, if I'm not exercising. So, I have a bit of a thing that I want to be doing something every day. And on the days when I'm feeling sick from the treatment, I find it psychologically frustrating as well as physically frustrating to not be able to…even go for a walk or have the strength to do anything like that. So, exercise has been part of my daily routine for a very long time. So I think it's…the change of lifestyle as much as how your body feels for not doing exercise. – ID #11
1.2 Increased Awareness of Illness	Well, it's hard to gauge, you know, where the disease starts and stops. You know, where it's just constant and symptoms. – ID #3
	It's like, it's like a bit of a black spot, you know… you can't do something that you want to do. And you know what the feelings are when you're able to do a particular exercise for a particular period of time, you know how you're gonna feel. But I just know that my system at the moment just wouldn't handle what I would do. Mentally, I think it wears on you and…then you keep saying to yourself, “Well, I wonder whether I'm ever gonna go back to what I had?” – ID #4
**2. Exercise Offers Hope**	
2.1 Reclaiming Control	I want to do stuff. I want to get myself as fit as I can and…not die just yet. – ID #10
	I'd like to maintain at least my level of activity that I have now. I don't want it to deteriorate anymore. I would like… for as long as I can. I realize that I only, I can do this for a certain length of time. But I want to try and keep going as long as I can. – ID #7
2.2 Cancer and Treatment-specific Benefits	I think it's important just to kind of keep your physical health at a point, at a good point where you can actually deal with side effects. So, I mean… immunotherapy is my first…treatment that I've experienced. So, I haven't done chemo. I haven't done any other ones. So, my, in my mind, it's like… I want to prepare myself for possible change in treatment, different side effects. I mean, your body, medication and your body sometimes is just unpredictable. So, my, my idea is just more to prepare myself, but also feel like I'm contributing to my own health. – ID #2
	No, I think it's totally important to have exercise because it's getting all the chemo around you, your cells and your body through, through your blood. And but also, it's not wasting your muscles away. And, and, and especially my bone density, density and my, and all my bones need strengthening…so it's really important. – ID #19
2.3 An Emotional Outlet	So getting out there and doing a bit of exercise takes your mind off, off your diagnosis as well. And if it makes you feel better, well, naturally, your body's gonna start feeling better as well, you know? – ID #14
	Well, well, [exercise], it lifts my mood. It, it levels me out. It levels things out. It makes me clearer. I can think clearer. – ID #1
**3. Exercise Barriers are Multifaceted**	
3.1 Symptoms are Burdensome	For a while there, it was sort of difficult, just because of not having muscle mass and just also at the moment, because of the breathing. It's a bit of an issue because breathing isn't as good as it used to be. So, I'm aware of my breathing and then I get sort of, if I exert myself really quickly, I can't breathe. – ID #8
	You know you tell yourself, I want to go for that walk. But you certainly cannot actually get yourself out of bed. It's the strangest feeling…kind of, it's just weird. Sort of, it's sort of almost…something to do with your will to get up and do it. You can't will yourself. I don't know if it's because of the tiredness because it messes with all the things in your body, and your brain. I don't know, I don't know what it is, but it's weird. – ID #20
3.2 Safety Concerns Reduce Confidence	I'm just worried that I'll be damaging the spine. That's the only thing, I think. Whether I'll get another crushed vertebrae… because I don't know why. – ID #16
	The exercise, I sometimes get a bit paranoid about whether it's the stiffness that I'm getting, because I'm not using [those] muscles…Yeah, so that's where I've got to sort of weigh out whether it is pain from building my muscles up again or whether it's pain from the fractures or, you know, my bones. I suppose I am a bit, bit worried, conscious of all that, because they're the, the tumors are in all my bones. – ID #19
3.3 Having Cancer in a Pandemic	Because of the COVID, I haven't been anywhere. I haven't been out. – ID #7
	Um, I don't think this, this lockdown thing helps at the moment, you know, because you can't see anybody, you can't really go. You know, I would, you know, my daughter would come and take me and she'd make me go for walks and things like that. – ID #6
3.4 Lack of Personalized Exercise Advice and Services	I think they may have responded to a question I asked about, "Is [exercise] okay?" But it has never been offered as a strategy to use to alleviate some of the symptoms I've experienced. – ID #11
	I think one of the frustrations I've been having is that these programs or physiotherapy, or going to the gym… there's no structure or continuity to them. I can't really track so much my progress or create milestones. There's no one really accompanying my physical rehabilitation. It's all just there's some exercise here, some exercise there, and you know, wish for the best sort of thing. – ID #13
3.5 External Factors	And having a one-on-one, like having a personal trainer, I would say financially because I don't work, and I don't have a pension. So, you know, it's just money out of your own savings, really. – ID #9
**4. Exercise Access and Support are Important**	
4.1 Professional Supervision and Structure are Valued	Oh, I know that [exercise] would be something that would be beneficial. And I've often thought, geez, maybe I would exercise if I could just do it one-on-one with someone. And if like, they could help me build myself up slowly, rather than…every time you see people exercising, [they’re] just like going at 100 miles an hour and it's like, well, that's not…what I could manage. Anyway, I would need someone who could slowly help me get to a better point. – ID #8
	“Well, [an exercise] program motivates me, a specific program.” – ID #1
4.2 Social Support is Motivating	I'm probably more motivated with the people around me doing it as well. Whereas, if I'm on my own, you don't need to do it. – ID #6
4.3 Access to Facilitate Exercise	But if you're going through chemo, traveling is a big thing. You want things nearby…It's got to be accessible because if you 're sick and you sleep half of the day, your only window is say, the afternoon. What are you going to do? You want to maximize the time you have to feel that…you have achieved something for the day as well. – ID #20

### Theme 1: Life is disrupted by cancer and cachexia

Participants provided diverse descriptions of profound physical, psychological, and social impacts of their cancer diagnosis and the experience of cachexia. Notably, being less physically active or having to give up certain physical activities due to a decline in physical health, was difficult for some participants to discuss and could be a source of distress. Others more openly reminisced about physical activities they previously enjoyed doing either in younger years or prior to being diagnosed. A change in physical condition, coupled with relinquished physical activities, roles, and responsibilities appeared to underpin a change in self-perception and enhance participants' awareness of being ill, creating a new, and often unsettling, normal.

#### Theme 1.1: Altered sense of self

Participants had an acute awareness of changes in their physical condition and health, which impacted participation in various events or physical activities, including occupational, household, social and exercise-based. The idea that “what we do” is closely linked to “who we are” was apparent. Grappling with a loss of independence and giving up roles and responsibilities, such as looking after grandchildren, were discussed. Some participants were grieving a prior sense of self. One participant (ID #9) described how they had changed “from being a very independent person to someone that relies on others” and accepting this change was an ongoing process. Changes in both physical function and reductions in physical activity, including both incidental physical activity and exercise, had wide-ranging implications. Negative feelings of sadness, frustration, boredom, and guilt were commonly expressed. One participant (ID #3), for example, described feeling guilty about not being able to walk their dog. A change in physical function also had specific social effects for some participants, including for one participant (ID #17) who expressed disappointment in no longer participating in their social walking group because they felt they couldn’t keep up with the group anymore. An exception was one participant (ID #19) who had previously taken part in triathlons and been highly physically active prior to their diagnosis of metastatic cancer. They explained how they didn’t miss long bouts of exercise training, simply because they were too tired and in too much pain in their current situation. This participant still had enough physical capacity to go on leisurely walks with friends and highlighted:

“I'm just pleased that I'm here and that I can still do what I can do, really.” – ID #19.

Thus, the type and extent of physical function changes and the restrictions they impose may relate to differences in the emotional responses elicited. Physical limitations with greater social repercussions or that impede autonomy may more adversely affect one’s sense of self and be particularly challenging to navigate.

#### Theme 1.2: Increased awareness of illness

The physical signs and symptoms of cachexia were often perceived as a manifestation of having cancer. Many participants commented on looking and feeling different, such as feeling frail or perhaps looking more like “a person with cancer” in a stereotypical sense. An increased awareness of being ill sometimes brought into question whether participants might ever go back to feeling like their normal selves again. Turning points of physical change along the cancer treatment continuum were discussed and coincided with an increased awareness of being ill. Often the increased awareness of their illness coupled with the onset of cachexia. Noticeable body weight loss, physical deconditioning, or exacerbated symptoms could be pinpointed concurrent to undergoing a certain cancer therapy or after being hospitalized, for example. A couple participants (ID #12 and ID #15) had also had previous cancer diagnoses and reflected on how they had “felt better” then and were able to physically do more, including exercise, when their cancer was considered early-stage. These participants had cancer recurrences and reflected on how their current situation felt dramatically different because of their weight loss and symptoms, which were perceived as signs of disease progression.

“So, I was still able to do normal things back then. This one… hit me a lot harder with the weight, I guess. Because with the liver, it's always different when it comes with the liver.” – ID #15

The increase in cancer symptoms could be a constant or prominent reminder of one’s deteriorating health. One participant discussed noticing their skeletal muscle mass declining and how this seemed to correlate with feeling more fatigued and breathless. The change in symptom burden increased their awareness of being ill. They said:

“It wasn't that long ago that I was asymptomatic and now, I'm symptomatic. So, you know, it does…sort of make you think…there is something going on inside me…you feel different... you're more conscious of the fact that…you are ill.” – ID #18

### Theme 2: Exercise offers hope

Regardless of each participant’s current individual level of physical activity, exercise was commonly perceived as beneficial for one’s health and wellbeing. For participants who were currently exercising, exercise participation was associated with feelings of accomplishment and pride. For participants who were less physically active, there was a divide between people who felt they were simply “not up to it” and those who were open to exercising more because they believed exercise was something they could do to help themselves. Indeed, exercise was often perceived as a possible antidote to some of the disruption or turmoil in participants’ lives that had been caused by their cancer. Taking part in exercise, or even perhaps the act of discussing exercise, kindled a level of hope.

#### Theme 2.1: Reclaiming control

Some participants discussed utilizing exercise as a current or potential tool to reclaim control in their lives. Exercise was perceived as something participants could do to actively contribute to their physical and psychological health in a time when their health could largely feel beyond control.

“For me, [exercise is] really important to have some control over my life.” – ID #11.

Interestingly, for one participant who described never regularly exercising prior to their cancer (ID #2), they now believed exercise to be part of their “survival mode.” Their diagnosis was described as a “life-changing moment” and presented them with an opportunity to reflect on what they could do for themselves to take back some control over their health. From a psychological perspective, participating in exercise could be empowering. One participant (ID #5) described how exercise was their “personal stand” against their diagnosis and a way to reclaim control over their psychological wellbeing.

“I don't want to think the cancer is beating me.” – ID #5

From a physical standpoint, exercise often presented as a positive opportunity for goal setting, including sustaining one’s physical function or capacity to live life more meaningfully by being able to still “do stuff.” While cancer and its treatment could strip away one’s strength, for example, exercise was a perceived strategy to try to regain what had been lost or prevent further physical deconditioning. Further, for participants who were maintaining a higher level of physical activity, they saw staying physically active or exercising as a personal responsibility.

“If you don't do it, you lose it.” – ID #19

#### Theme 2.2: Cancer- and treatment-specific benefits

The prospect of exercise acting as a supportive care strategy to better manage one’s cancer diagnosis was often a source of motivation. Particularly among participants who were maintaining some level of physical activity, exercise was identified as a strategy to alleviate common cancer symptoms. A few participants made comments about how exercise, often walking outside, could improve symptoms, such as fatigue, nausea, and appetite loss. While less tangible, a handful of other participants also discussed their hope for exercise to improve their treatment tolerance, treatment efficacy and even their prognosis.

“I think [exercise], well, I'm hoping, it's going to help you improve, get better results for your tests. I know mine's incurable…but it might extend time.” – ID #9

Most participants were not familiar with the term cachexia or elements of the syndrome. Yet, several commonly described their belief that exercise may address some of the adverse effects of their cachexia, including regaining muscular strength. No participants expressed significant concerns about exercise exacerbating weight loss. Some mentioned that exercise was once a strategy to lose weight prior to their cachexia and reflected on how their exercise goals had shifted towards maintaining strength and physical function and away from losing weight. One participant (ID #18) highlighted their hope for exercise, including resistance exercise training, to help manage or reverse their weight loss. In a few cases, the physical health, cancer, and treatment-specific benefits of exercise were reinforced by healthcare providers through discussions or direct referrals to cancer exercise professionals, including exercise physiologists or physical therapists. Medical advice to exercise and encouragement from healthcare providers could be a source of motivation for some participants.

“The surgeon who originally diagnosed me, he said, ‘Get to a gym right now and start building your muscle strength.’ So, I did.” – ID #12

#### Theme 2.3: An emotional outlet

Exercise was viewed as a tactic to relieve psychological distress and improve mental and emotional wellbeing. Some of the positive mental benefits of exercise that were described included exercise’s ability to help participants maintain a positive outlook or as one participant phrased (ID #15), “it puts you in a better place.” Similarly, another participant (ID #14) described how sitting around and doing nothing at home could make them “dwell on the situation a bit more.” A common talking point included how one’s diagnosis, and the underlying uncertainty for the future, had the potential to make participants feel like a “victim” or “down in the dumps.” Exercise was thus seen as an important way to overcome negative feelings by improving one’s train of thought or allowing participants to feel more like their “normal selves.”

“I think [exercise] gives you more confidence in yourself." – ID #12

Spending time exercising outside, with others, or simply away from home was also believed to reduce the feeling of being confined by one’s cancer or could act as a positive distraction. Exercise was perceived as a positive opportunity to reflect and gain perspective.

“[Exercise can be] a really good…mental break from home…and then you see…there's a bigger world out there than…what you're going through.” – ID #2

### Theme 3: Exercise barriers are multifaceted

Each participant described struggling with multiple, complex exercise barriers that directly related to or were often exacerbated by their current cancer diagnosis. Challenges and frustrations with not feeling well enough to exercise, despite wanting to exercise or acknowledging the importance of exercise, were expressed. Multifaceted exercise barriers prevented participants from experiencing the full extent of the physical and psychosocial benefits that they perceived exercise could offer. Exercise barriers appeared to hinder the frequency, type, duration, and intensity of exercise as well as apparent exercise self-efficacy.

#### Theme 3.1: Symptoms are burdensome

Cancer and treatment-related symptoms were wide-ranging, debilitating, and common across all participants. Most participants dealt with several concurrent symptoms, such as fatigue, gastrointestinal symptoms, shortness of breath, and pain, that interfered with exercise motivation and perceived physical capacity for exercise.

“Well, if you're in one of those troughs of fatigue, then you don't feel like exercising at all.” – ID #10

Many participants discussed having to self-manage their symptoms by reducing their exercise to avoid overexerting themselves. Some discussed needing to adjust the timing and duration of exercise during periods where symptoms felt worse. While several participants understood that exercise could improve some of their symptoms, many still struggled with motivation to exercise when they were feeling particularly unwell. Further, when participants’ awareness of the possible role that exercise could play in symptom management was lower, symptoms appeared to be a larger exercise barrier. In these instances, participants felt they would only be able to start exercising, if their symptoms were to significantly improve. As one participant struggling with fatigue and breathlessness stated:

“If my physical situation improves, I will be a bit more active. It's as simple as that.” – ID #3

#### Theme 3.2: Safety concerns reduce confidence

Several participants raised exercise safety concerns relating to their existing cancer diagnosis or additional comorbidities. Particularly among older participants and one participant with brain cancer, there were balance concerns and a fear of falls. Two participants (ID #9 and ID #16) occasionally used mobility aids (i.e., cane and four-wheeled walker, respectively) outside of the home or for exercise, such as short walks. Specific fears about performing activities around the house (e.g., showering) among those with balance concerns were also discussed and this translated into some fears about performing specific movements while exercising. One participant expressed how a previous fall scared them and reduced their confidence:

“I think it scared, the fall, definitely scared me. I didn't realize and then I had a couple of falls, like, in the bathroom…and it scared me.” – ID #7

Other exercise safety concerns included the perceived risk of bone fractures or exacerbated pain with increased bone fragility and the presence of bone metastasis. In addition, participants with greater comorbidities, such as cardiovascular disease or osteoarthritis, also expressed concerns about exercise exacerbating their conditions. Most safety concerns resulted in fear or reduced confidence exercising alone. Moreover, others were hesitant about stepping into traditional fitness centers, where the exercise options may not be tailored to participants’ baseline health status and therefore, be potentially unsafe.

“I don't think in a lot of cases [fitness centers] are fitting to people who have got different conditions.” – ID #4

#### Theme 3.3: Having cancer in a pandemic

The COVID-19 pandemic and associated public health restrictions greatly reduced opportunities for exercise. City or state-wide lockdowns often prevented participants from leaving home. For some, this meant current exercise opportunities had been eliminated. One participant (ID #1) discussed how their regular yoga class was cancelled during lockdown and they were not motivated to seek out online classes or services. For others, being forced to spend more time at home decreased physical activity motivation entirely. The pandemic reinforced or normalized sedentary behavior, as staying at home was perceived to be the “right thing to do.” Participants also expressed a fear of contracting the COVID-19 virus, particularly given their immunocompromised status. One participant described how the fear of becoming ill with COVID-19 influenced their willingness to visit public spaces, including fitness centers:

“It's just COVID's changed everything…I'm just paranoid about going in with people around and [it’s] changed my whole mindset of going into a gym.” – ID #19

#### Theme 3.4: Lack of personalized exercise advice and services

A disappointment towards a lack of personalized or cancer-specific exercise advice and services as a part of their standard care was expressed strongly by a handful of participants. Many also assumed their healthcare providers were aware of their current level of physical activity, without ever having discussed it in detail. Another participant discussed having to take  initiative to ask their healthcare provider about exercise services, which did eventually lead to a referral to an exercise physiologist:

“Nobody really said anything. It was, it was actually me that brought [exercise] up because I said that I'd lost so much weight and I've lost all my muscle tone.” – ID #14

In other cases, participants reported asking specific questions about exercise but felt discussions with healthcare providers were too brief or generic. The lack of recommendations led to frustration, particularly among participants who were motivated to exercise but were perhaps struggling with a lack of knowledge about what exercise to do and how to do it safely. Participants discussed receiving little feedback or vague statements from their treating healthcare providers, such as “do what you're comfortable with.”

“You get, you get very little back from them. It's disappointing, to be honest.” – ID #10

#### Theme 3.5: External factors

Weather, finances, and family responsibilities also interfered with participants' ability to exercise or willingness to seek out exercise services. Notably, symptoms, balance concerns, or functional limitations associated with one's cancer diagnosis or comorbidities could exacerbate the extent to which common external factors interfered with exercise participation. While bad weather may have been an exercise barrier prior to some participants’ cancer diagnosis, additional safety concerns associated with exercising outside, such as rain and slippery footpaths, now amplified the extent to which bad weather might restrict their exercise. Furthermore, physical deconditioning also meant that some participants could no longer drive or confidently use public transportation. Thus, the time and effort needed to arrange transportation (e.g., arranging for a carer to drive them somewhere) made attending regular in-person exercise appointments impractical.

“I still depend on [my spouse] to take me around to places. So, it's not like I could just get to the gym by my own.” – ID #13

### Theme 4: Exercise access and support are important

Exercise preferences differed between participants and were frequently based on past physical activities and experience. Participants often went back and forth between describing their exercise preferences *prior* to their cancer diagnosis and then reflecting on how they were no longer able to do what they previously could. Existing exercise preferences tended to be heavily entwined with confidence in current physical abilities, but also convenience. Many participants described walking as their main form of exercise, as it aligned with their current physical capacity and was accessible. To improve physical activity levels and allow participants to reap greater benefits from exercise, several factors were perceived to help overcome common exercise barriers. Specifically, participants described the importance of engaging in structured exercise programs, receiving supervision from exercise professionals, having social support to stay motivated, and selecting convenient exercise settings.

#### Theme 4.1: Professional supervision and structure are valued

Access to both professional supervision and programmed or “structured” exercise was perceived as necessary to safely exercise and maintain exercise motivation. Participants discussed the importance of working with an exercise professional who had an awareness of their medical history and could prescribe personalized exercise that felt safe and tolerable. For some participants who felt weak or physically deconditioned, they discussed wanting reassurance from an exercise professional that the exercise would not feel too challenging and strenuous. Other participants felt professional supervision would also help overcome safety concerns, such as concerns regarding musculoskeletal injuries or fractures due to the presence of bone metastasis.

“So just I think the concern was that I'm not doing it properly. That's why I probably would want somebody there to say, ‘Oh, you know, you should be doing this.’” – ID #16

Several participants also emphasized the importance of setting an exercise routine to keep them motivated, accountable, and to ultimately, establish behavior change long-term.

“I think sometimes when… you’re scheduled to turn up type of thing, you can't back out.” – ID #10

Setting a fixed exercise routine or committing to an exercise program with some level of supervision (i.e., fully supervised or partially supervised and partially self-guided) could ease the pressure of having to plan the type and timing of ones’ own exercise. Some participants had previously engaged in structured and supervised exercise, including prior to the COVID-19 pandemic or prior to their cancer diagnosis, and reflected on the importance of building exercise into their weekly schedule to facilitate exercise adherence. When COVID-19 resulted in the cancellation of one participant’s structured exercise program, they mentioned struggling to start exercising again:

“I need to get back into the motivation, like, it's there in my mind…I know that I need to do it. It's just, I think, setting a routine.” – ID #15

#### Theme 4.2: Social support is motivating

Social networks, including friends and family, were perceived as important sources of exercise motivation. One participant (ID #4), for example, discussed how their spouse was a source of encouragement to go for regular walks. Past or current preferences for group-based exercise or exercise that doubled as a social activity were also discussed. Exercising with others for some participants made exercise inherently more enjoyable. One participant (ID #14) also mentioned that walking with a small group rather than on their own could make exercise feel safer, such as in the event they felt breathless and needed a rest. Social and emotional support and a sense of normalcy were also noted as potentially valuable components of opportunities to exercise alongside other people with cancer. When asked if they would prefer to exercise with friends, family, or in a setting with other people with cancer, one participant said:

“Other people who have a similar diagnosis because we probably can be all rubbish together.” – ID #7

Most participants had not been offered the opportunity to take part in a cancer-specific group exercise program but expressed an openness to it and reflected on perceived psychosocial benefits. One benefit was the chance to share an experience with other people facing similar challenges.

#### Theme 4.3: Improved access to facilitate exercise

Participants preferred convenient exercise options and locations. Strategies to increase access to exercise opportunities, including locating exercise facilities closer to home or offering home-based exercise, were believed to facilitate exercising more regularly. One participant (ID #13) mentioned that exercising at home would “allow me to do it more often.” Convenient exercise options were perceived to help overcome common external factors that were barriers to exercise, but also could allow participants to make better use of their time and not waste mental and physical energy traveling. When prompted about exercise support offered via telehealth, several participants expressed that its convenience was a clear asset and could be a way to increase the accessibility of supervised exercise.

“Well, if [telehealth] makes [exercise] much more available, then it's, that's the greatest benefit.” – ID #1

However, in-person exercise support was often still predicted to be superior in-terms of being physically assessed by an exercise professional, receiving exercise-related feedback, and having access to exercise equipment and space.

“I would always prefer face-to-face, if given a choice.” – ID #5

## Discussion

The current study sought to understand the perceptions of exercise in a diverse group of patients living with cancer cachexia and advanced or metastatic disease. Participants richly described the impact of their diagnosis, cancer treatment, and cachexia on their current ability to be physically active, which had broad effects on their lives. Despite commonly reporting reductions in physical activity and function, exercise was still viewed as beneficial and appeared to instill feelings of hope. Challenges planning and completing exercise were common. Each participant described unique barriers to exercise, including the considerable burden of their cancer symptoms. To improve exercise participation, participants highlighted the importance of exercise support and access. Support was discussed in the form of (personalized) professional exercise supervision, structured exercise, and socially, via planned group-based exercise or exercising within social networks. Increasing exercise access (e.g., through more convenient exercise opportunities) was also perceived to facilitate exercise.

Current physical activity levels varied between participants. Several participants expressed a reduction in their physical activity, including exercising less often compared to prior to their diagnosis or not currently being able to exercise at all. Physical activity levels typically decrease and remain low following a cancer diagnosis [47] and are low among people with advanced cancer [48] and cancer cachexia [49]. Among 196 outpatients with cancer cachexia who completed surveys, patients reported low levels of physical activity and low exercise self-efficacy, with beliefs that even moderate intensity exercise may be too difficult [49]. Our findings corroborate these data. We found participants’ diagnoses and cachexia disrupted their lives on several dimensions, physically, mentally, emotionally, and socially, including their ability to be physically active. Changes in lifestyle and independence were described by participants, leading to an altered sense of self. Moreover, the confronting physical signs and symptoms of cachexia, including diminishing physical function, were perceived as a manifestation of one’s illness. Other studies also report that involuntary weight loss, worsening symptoms, or additional signs of disease progression, are a source of distress among people with cancer [14, 50]. The experience of having advanced cancer and cachexia evidently involves physical health changes, but also psychosocial dimensions, including alterations in sense of self and living with an increased awareness of one’s illness.

Exercise was often perceived to ignite hope and was recognized as important for physical health and wellbeing. Hope is a complex and important construct in palliative cancer care and there is a dynamic relationship between hope and coping [51]. Hope has been presented as traversing from “a faint glimmer or glow” to “a spark, flame, or fire” and can change depending on circumstances, such as bad or good news, or symptom progression [52]. We found exercise, or conversations around exercise, may stimulate hope to overcome the disruption to participants' lives that had been caused by their cancer and to focus on positive events and opportunities for the future. Firstly, exercise was described as a way to reclaim control over one’s health. A recent qualitative study in men with metastatic prostate cancer reported similar findings among active participants who associated exercise with re-establishing control, for example, by feeling they were participating in their own care [53]. Multiple participants also discussed the hope for exercise to provide cancer- and treatment-related benefits, including improving cancer treatment effectiveness, alleviating symptoms, and extending survival. Another qualitative study in metastatic lung cancer patients also reported that participants expressed that exercise instilled hope for surviving their cancer [31]. Further, we found exercise acted as an emotional outlet and opportunity to take one’s mind off their diagnosis. Often, the psychological benefits of exercise seemed more palpable. Participants could associate immediate feelings of improved mood, for example, with a single bout of exercise rather than perhaps physical changes, which may be slower to experience and require participating in regular exercise over a longer period.

Participants did not express explicit concerns about exercise exacerbating their cachexia (i.e., weight loss). Instead, exercise was viewed as a tool to address various dimensions of the cachexia syndrome, including building skeletal muscle mass and strength, improving appetite, and managing weight loss. A prior qualitative study found that one participant with advanced cancer who had experienced 30 kg of body weight loss held concerns about exercise exacerbating their weight loss [33]. Cachexia is a syndrome spanning from pre-cachexia, to cachexia, and then refractory cachexia [1]. Relative to patients with more extensive weight loss (refractory cachexia), rapid disease progression, and an expected survival < 3 months, participants in the current study had earlier phases of cachexia (average weight loss 6%). Most participants were aware of their muscle mass and weight loss and in some cases, had adjusted their attitudes towards exercise as a weight loss strategy. Instead of exercising to lose weight as they might have done previously, exercise was now perceived as a tool to build strength, sustain physical function, and live life more meaningfully.

Several considerable barriers to exercise were identified in our study. Diverse cancer symptoms, including intense fatigue, influenced perceived exercise capacity and motivation. Cancer symptoms are a well-established exercise barrier among people with advanced cancer [32–36, 48] and in patients with cachexia, symptoms occur in greater numbers and with worse severity [29]. Optimizing the medical management of symptoms and educating patients on the direct role exercise might play in improved symptom management, may stimulate patients’ interest in exercise. Palliative patients with cancer cachexia who receive multidisciplinary care, including consultation with a palliative care physician, nurse practitioner, dietitian, and physical therapist, report improvements in cancer symptoms, including fatigue, pain, anorexia, and nausea [54]. Such multimodal treatment approaches may be an important first step to manage symptoms prior to prescribing structured exercise, particularly among inactive patients or those with lower exercise motivation. Exercise safety concerns were also a notable barrier, including concerns relating to balance or falls, bone fractures (with bone metastasis), and exacerbating pre-existing musculoskeletal or other comorbid conditions. Particularly among older adults (≥ 65 years), balance concerns, physical limitations, and managing other comorbid conditions may be more prominent exercise barriers [33]. Individually tailored exercise prescription approaches may therefore need to initially include balance training or low intensity exercise to improve patients’ confidence in their physical abilities and overcome safety concerns.

Limited in-depth discussions with healthcare providers about exercise was a source of disappointment and potential reason for lower exercise engagement for some participants. A prior qualitative study of patients with advanced lung cancer also reported that conversations about exercise between patients and their healthcare providers were infrequent or too generic [27]. Our participants reported mixed experiences. When healthcare providers discussed exercise or made referrals to exercise-services, this could be motivating, reinforcing patients’ positive beliefs about exercise. When discussions were brief or no direct recommendations were provided, participants sometimes expressed feeling frustrated. Healthcare providers acknowledge that exercise is important for people with advanced cancer, although concerns relating to exercise in patients with bone metastases, including an increased risk of fractures, have been noted [45]. The greater health complexities of an advanced cancer diagnosis may interfere with the nature of conversations that healthcare providers have with their patients about exercise. Current information on healthcare provider attitudes towards exercise for cancer cachexia is also more limited. However, elements of the cachexia syndrome, such as involuntary weight loss, may increase clinical concerns about recommending exercise to this patient group and may underpin differences in exercise recommendations provided to patients with and without cachexia.

Multiple significant external factors appeared to influence current exercise habits. The most notable being the COVID-19 pandemic, which not only directly eliminated exercise opportunities during periods of lockdown, but also introduced health and safety concerns. Systematic review evidence suggests the COVID-19 pandemic and subsequent lockdowns in 2020 were associated with significant reductions in physical activity levels and increased sedentary behaviour worldwide, including among adults living with chronic disease [55]. Moreover, COVID-19 has greatly affected the psychological health of people with cancer, including due to fears around contracting COVID-19 and being immunocompromised [56]. We found that such concerns may impact exercise motivation and preferences long-term, including willingness to visit public exercise facilities. Lastly, other external factors, including weather, travel, and cost, were reported exercise barriers and are well-documented barriers among people with cancer [57]. Ongoing and future exercise recommendations and programming should continue to consider external or logistical factors that influence exercise interest and uptake, most notably the possible lasting effects of the COVID-19 pandemic.

To foster regular exercise participation, participants expressed a desire for greater exercise support and access. Professional exercise supervision with a cancer exercise professional was often desired to ensure exercise was safely prescribed, monitored, and progressed. Preferences for establishing an exercise routine or engaging in structured exercise to increase exercise motivation, accountability, and adherence, were also mentioned. Social support, including in the form of group-based exercise, may also increase exercise motivation and inherently make exercise more enjoyable. Patients with advanced cancer who have engaged in professionally supervised, structured exercise interventions with other people with cancer often report positive experiences [58–62]. Indeed, prior research suggests physical activity or exercise interventions among people with cancer can provide opportunities to build relationships and provides a shared experience that is positive [63]. Our findings support that exercise interventions specifically for people with cancer may offer social benefits, as participants discussed their potential to foster social connectedness and create a sense of normalcy. Many participants also concurrently discussed wanting more convenient exercise options, including home-based exercise. Other studies in palliative cancer settings [36, 64], including one study in cancer cachexia [49] report similar patient preferences for home-based exercise and exercise that is lighter intensity. Leisurely walking was the most common type of exercise that was  reported among participants in the current study. Thus, while many reported preferences for more structured, supervised exercise, lower intensity exercise that can be completed at home or close to home may be more feasible and tolerable.

### Strengths and limitations

The current qualitative study provides novel findings on the perceptions of exercise among patients with advanced cancer with cachexia. Participants were drawn from real-world clinical settings and our sample was diverse in terms of participant sex, age, cancer type and treatments, and physical activity levels, which increases the breadth of our findings. We did not, however, quantify symptoms or performance status, which may limit the transferability of our findings. We also only included patients who could communicate in English and thus, findings may not extend to non-English speaking patients. Our patient sampling may also be vulnerable to selection bias. Patients, for example, with a greater underlying interest in exercise may have been more willing to participate in an exercise research study. However, only two participants declined to be interviewed and the study sample included a balance of both physically active and inactive participants, suggesting our findings are trustworthy. The low decline rate also suggests that even physically inactive participants were willing to discuss their exercise habits and experiences. A further limitation is that we did not perform repeat interviews due to the potential increased burden on participants’ time and vulnerable nature of our sample. However, repeat interviews may have provided a richer understanding of participant perspectives and experiences explored in our study.

### Directions for future research and clinical implications

Preliminary findings from our study can be used to inform future research studies on the topic of exercise for people with advanced cancer and cachexia. To broaden our understanding of patient perceptions of exercise, further qualitative research on novel concepts we identified may improve our understanding of patient experiences. An example includes further exploration of the meaning of physical function changes and functional-related goals, particularly among participants with established functional impairment. Moreover, what exercise means among palliative and nonpalliative healthcare providers and what role it may have in supporting patients with cancer cachexia has been underexplored and is an important topic for future research studies. A more precise quantitative characterization of symptom burden, physical fitness and function, muscular strength, and body composition (e.g., skeletal muscle mass) coupled with a qualitative research component in a mixed methods study may also add important new knowledge. This study design could improve our understanding of patient experiences as they relate to key physical health metrics with the potential to be modified with exercise. Our findings suggest there are patients with advanced cancer and cachexia who are open to and willing to participate in supervised and structured exercise interventions. Thus, the next crucial steps include building a stronger evidence base to establish the feasibility and efficacy of supervised and structured exercise as either a stand-alone intervention or as a part of a multimodal approach in patients with advanced cancer and cachexia through randomized controlled trials. From a clinical perspective, we encourage healthcare providers to discuss exercise, including its potential benefits, with palliative patients who may have pre-cachexia or cachexia and consider referral to oncology specific exercise services, if accessible and medically suitable. To foster exercise participation, special attention should be paid to symptom burden and current clinical management, as well as addressing underlying safety concerns associated with any functional limitations or comorbid conditions. Exercise intervention design for clinical settings or in future research studies for patients with advanced cancer and cachexia may wish to consider offering structured, supervised and group-based exercise at convenient locations or at patients’ home. Combining unsupervised home-based with supervised exercise, which may include incorporating telehealth, may help balance patient exercise preferences that we identified in the current study.

## Conclusions

The aim of this qualitative study was to capture the perceptions of exercise among patients with advanced cancer and cachexia. Our findings suggest an advanced cancer diagnosis and the experience of cachexia intensely disrupt patients’ lives, including their ability to be physically active. Despite these challenges, exercise could ignite a level of hope in participants to take back control and better manage their health and wellbeing. Barriers to exercise were multifaceted, but included living with burdensome cancer symptoms and the overwhelming impact of the COVID-19 pandemic on exercise opportunities. To facilitate exercise, participants discussed preferences for professionally supervised and structured exercise, social support, and convenient exercise options. Our study findings can be built upon and explored further in future research to improve and refine exercise recommendations and intervention design for patients with advanced cancer and cachexia.

## Supplementary Information

**Additional file 1: Table.** Initial Coding Framework.

## Data Availability

A portion of the data is presented in Table 3 and the written text within the Results section. Due to the sensitive nature of the interview transcripts, data are not publicly available. Additional information may be made available upon request by contacting the corresponding author.
