# Supplementary material for: “I want to get myself as fit as I can and not die just yet” – Perceptions of exercise in people with advanced cancer and cachexia: a qualitative study
Source: BMC Palliat Care. 2022 May 17;21:75. doi: 10.1186/s12904-022-00948-x (PMC9110215; doi:10.1186/s12904-022-00948-x)
Supplement: Supplementary file 1 — Additional file 1: Table. Initial Coding Framework. [file 12904_2022_948_MOESM1_ESM.docx]

***Table*** *Initial Coding Framework*

| Categories and Descriptions | Sub-categories | Codes |
| --- | --- | --- |
| Current Exercise and Physical Activity  Current exercise and physical activity, including incidental or non-leisure based physical activity and planned, structured exercise. | Non-Leisure Physical Activity | Gardening  Household-based (e.g., Cleaning)  Other (e.g., Transportation) |
|  | Exercise | Walking Outside  Strength Training  Other (e.g. Yoga) |
| Changes in Activity and Function  Reported changes in physical function and ability to move since being diagnosed with cancer, including the ability to engage in specific types of physical activity, and the perceived psychosocial impact. | Physical Changes | Relinquished Physical Activities  Physical Deconditioning  Turning Points of Change in Function |
|  | Psychosocial Impact | Acceptance/Awareness of Illness  Negative Emotional Response to Physical Changes  Expression of Unmet Clinical Need |
| Positive Beliefs about Exercise  Perceived importance of and benefits associated with physical activity and exercise including, physical, mental, emotional, and social wellbeing. | Physical Aspects | Has Cancer-specific Benefits  Improved Physical Health |
|  | Psychosocial Aspects | Positive Mental or Emotional Effects  Exercise as a Social Activity |
| Exercise Motivators  Factors perceived to currently support or *potentially* *increase* exercise and physical activity participation, including intrinsic and extrinsic motivators. | Contribute to Health and Wellbeing | Help with Cancer and Symptoms  Improved Mental Wellbeing  Get Stronger  “Feeling Better” |
|  | Structure and Support | Creating Structure and Routine  Community or Social Support  Professional Exercise Supervision  Discussion with Healthcare Team |
| Exercise Barriers  Factors perceived to currently limit or prevent exercise and physical activity participation. | Since Cancer Diagnosis | Cancer Symptoms (e.g., Fatigue, Nausea)  Treatment Schedules  Change in Motivation |
|  | Other Health-Related | Other Injuries and Illnesses  Concerns with Safety |
|  | Since the Pandemic | COVID-19 Restrictions and Risk |
|  | Logistics/Environmental | Travel  Family Responsibilities  Finances  Weather |
| Exercise Preferences  Thoughts about preferred exercise settings, types and time based on what was considered most enjoyable, interesting, and feasible in each participant’s current situation. | Setting | Outdoors  Close to or at Home  Fitness Centres  Telehealth |
|  | Type | Structured Exercise Training  Group-based or Classes  Walking  Individual Exercise  Open to Trying New Types |
|  | Time | Duration  Time of Day |
